# Optimising poly(lactic-co-glycolic acid) microparticle fabrication using a Taguchi orthogonal array design-of-experiment approach

**DOI:** 10.1371/journal.pone.0222858

**Published:** 2019-09-26

**Authors:** R. A. Mensah, S. B. Kirton, M. T. Cook, I. D. Styliari, V. Hutter, D. Y. S. Chau

**Affiliations:** 1 Department of Clinical and Pharmaceutical Sciences, School of Life and Medical Sciences, University of Hertfordshire, Hatfield, England, United Kingdom; 2 Department of Biomaterials and Tissue Engineering, Eastman Dental Institute, UCL, London, England, United Kingdom; Brandeis University, UNITED STATES

## Abstract

The objective of this study was to identify, understand and generate a Taguchi orthogonal array model for the formation of 10–50 μm microparticles with applications in topical/ocular controlled drug delivery. Poly(lactic-co-glycolic acid) (PLGA) microparticles were fabricated by the single emulsion oil-in-water method and the particle size was characterized using laser diffraction and scanning electronic microscopy (SEM). Sequential Taguchi L_12_ and L_18_ orthogonal array (OA) designs were employed to study the influence of ten and eight parameters, respectively, on microparticle size (response). The first optimization step using the L_12_ design showed that all parameters significantly influenced the particle size of the prepared PLGA microparticles with exception of the concentration of poly(vinyl alcohol) (PVA) in the hardening bath. The smallest mean particle size obtained from the L_12_ design was 54.39 μm. A subsequent L_18_ design showed that the molecular weight of PLGA does not significantly affect the particle size. An experimental run comprising of defined parameters including molecular weight of PLGA (89 kDa), concentration of PLGA (20% w/v), concentration of PVA in the emulsion (0.8% w/v), solvent type (ethyl acetate), organic/aqeuous phase ratio (1:1 v/v), vortexing speed (9), vortexing duration (60 seconds), concentration of PVA in hardening bath (0.8% w/v), stirring speed of hardening bath (1200 rpm) and solvent evaporation duration (24 hours) resulted in the lowest mean particle size of 23.51 μm which was predicted and confirmed by the L_18_ array. A comparable size was demonstrated during the fabrication of BSA-incorporated microparticles. Taguchi OA design proved to be a valuable tool in determining the combination of process parameters that can provide the optimal condition for microparticle formulation. Taguchi OA design can be used to correctly predict the size of microparticles fabricated by the single emulsion process and can therefore, ultimately, save time and costs during the manufacturing process of drug delivery formulations by minimising experimental runs.

## Introduction

Microparticles (MPs) may be designed to allow precise delivery of small quantities of potent drug and increase the local concentration of the drugs in the target tissue [1, 2]. MPs for use in drug delivery may be manufactured to provide controlled and sustained release, provide stability to the encapsulated drug and may reduce the side-effects of the drug [2–4]. The fabrication of MPs depends on the choice of suitable polymeric materials. An ideal polymeric material must be non-toxic, biodegradable and biocompatible [4]. In this work, poly(lactic-co-glycolic acid) (PLGA) was chosen for MP fabrication as it possesses the suitable properties, is readily available and approved by regulatory authorities (i.e. FDA) for clinical use in humans [5]. In the body, PLGA undergoes hydrolysis to generate lactic and glycolic acid which do not cause carrier toxicity or immunological response and explains the success of PLGA as a biodegradable polymeric material [6, 7]. When used for drug delivery, this hydrolysis leads to the degradation of the PLGA matrix and any drug incorporated within is liberated in a time-dependent fashion, typically over several weeks.

Various techniques are employed in the preparation of MP, including solvent emulsion evaporation (single or double), spray drying, polymerization, and phase separation. These techniques must fulfill certain standards which include the ability to achieve a controlled particle size and incorporate a required application-specific drug concentration [8–11]. Comparative studies have shown that the solvent evaporation technique, a simple method requiring only glassware and a magnetic stirrer is economical, convenient, robust and reproducible [12–14]. This technique can therefore be used to provide preliminary information regarding the parameters that influence the production of MP during scale up [14, 15]. The single emulsion method comprises of an oil in water (o/w) emulsion process. This method uses on a volatile organic solvent (e.g. dichloromethane (DCM); ethyl acetate (EAc)) containing dissolved polymer and the drug to be loaded. This organic phase and an aqueous phase containing a dissolved surfactant (e.g. PVA) are emulsified to create an o/w emulsion (primary emulsion). A surfactant is included in the aqueous phase to stabilize the organic droplets formed after the emulsification process. These polymer droplets harden due to removal of the organic solvent via dissolution and evaporation [16–20]. The process parameters involved in this method are known to effect the physical properties of the MPs produced, such as particle size and dispersity [21]. The MP size is important in determining the drug release profile and mode of application, such as the ability to pass through a needle. Furthermore, the particle size is vital with respect to tissue irritation [6, 22]. Hence, there is a need for careful selection of the process parameters in order to fabricate the most appropiate MP size.

Researchers have been able to identify several process parameters that influence the formulation of MPs by changing one variable at a time to achieve the desired particle sizes [23]. However, this optimization method is inefficient, and thus costly, and a systematic approach based on design of experiments (DOE) may be beneficial [23, 24]. The significance of optimization methodology and DOE in research and development has been demonstrated in several studies [20, 23–25]. DOE is a systematic technique used to determine the relationship between parameters affecting a method and the response produced by the process. The information allows the researcher to identify and select the parameters and levels which have significant effects on the final response/output. One such DOE methodology is Taguchi design [25].

Taguchi design is a statistical tool used to optimize parameters with complex interrelationship. This design ascertains that not all parameters that cause variability can be controlled. The design evaluates and identifies the controllable parameters that reduce the effect of the uncontrollable parameters (noise parameters) [24–26]. Furthermore, for Taguchi design, several parameters can be analyzed at the same time with few experimental runs. Taguchi design uses an orthogonal array (OA) to estimate the effects of parameters on the response mean and variation. An OA means the design is balanced so that parameter levels are weighted equally. Hence, each parameter can be assessed independently of all the other parameters, and the effect of one parameter does not affect the estimation of a different parameter. This makes it more economical and efficient than conventional experimental methods (for example the ‘one-parameter-at-a time’ method) [23, 26–30]. Additionally, Taguchi design uses signal to noise ratio (S/N ratio), a statistical measure of performance to evaluate the data for the responses. This S/N ratio serves a purpose of measuring how the response varies relative to the correct parameters and their optimal levels for the target value or range. The main goal of the experiment constructed with Taguchi design is to find the best settings of control parameters level involved in order to maximize the S/N ratio. Three available standard types of S/N ratios: the smaller-the-better, the nominal-the-best, and larger-the-better are employed in Taguchi design based on the desired response. The smaller-the-better is applied to minimise the response, the nominal-the-best to target the response and the larger-the-better to maximise the response [23–34]. The goal of this paper was to employ Taguchi OA designs to explore the effect of process parameters on particle size using the single o/w evaporation technique to obtain a drug delivery system for topical application.

## Materials and methods

### Materials

PLGA copolymers with lactic: glycolic acid ratios of 1:1 (MW: 29–38 kDa) were purchased from Evonik (Darmstadt, Germany). PVA samples: 98% hydrolysed (MW: 13–23 kDa), 99+ % hydrolysed (MW: 146–186 kDa), 99+ % hydrolysed (MW: 89–98 kDa), bovine serum albumin (~66kDa) and QuantiPro Bicinchoninic Assay (BCA) kit were obtained from Sigma-Aldrich (Poole, Dorset, UK). Anhydrous dichloromethane (≥ 99.8% with 50–150 ppm amylene as stabiliser) and ethyl acetate (≥ 99.5%) were purchased from Fisher Scientific (Loughborough, Leicester, UK).

### Experimental design

This study presents the application of Taguchi OA design to explore the effect of 10 processing parameters on particle size using the solvent evaporation technique. These parameters were: concentration of PVA, molecular weight of PVA, concentration of PLGA, type of solvent (i.e. DCM, EAc), concentration of PVA in the hardening bath, stirring speed, ratio of organic/aqueous phases, vortexing speed, duration of speed and time for solvent evaporation. The goal was to generate a formulation model to produce 10–50 μm PLGA MP which has been suggested to be to most suitable for topical and ocular applications. PLGA has a tailored biodegradation rate and this is dependent on the copolymer ratio. PLGA of different lactide/glycolide (copolymer) ratios; 50:50, 65:35, 75:25 and 85:15 lactide/glycolide are manufactured. The 65:35, 75:25 and 85:15 lactide/glycolide copolymers have extended degradation half-lives as compared to 50:50 lactide/glycolide copolymers that have the fastest degradation half-life of about 60 days [2, 5–7]. PLGA copolymers with lactide: glycolide ratios of 50:50 were utilized in this research due to the proposed end-point therapeutic application.

#### Selection of parameters and levels

In a typical solvent evaporation technique, an extensive number of processing parameters can influence the MP size (response). In this study, ten parameters deemed experimentally controllable that influence particle size were identified by critically assessing relevant literature and are listed in Table 1. Bible *et al*. [16] identified polymer concentration, vortexing speed and duration, speed of hardening bath and PVA concentration as possible important factors which affect particle size. Vyslouszil *et al*. [35] conducted the study of the influence of a stirring speed (600, 1000 rpm), PVA concentration (0.1%, 1%) and organic solvent (DCM, EAc) during the formulation of drug-loaded PLGA microspheres. Sharna, Madan and Lin [36] included homogenization speed, evaporation time, surfactant concentration, organic/aqueous phase ratio, PLGA polymer type and concentration as process parameters for the creation of paclitaxel-loaded biodegradable PLGA nanoparticles using modified solvent evaporation method.

**Table 1:** Parameters and associated levels used in L_12_ design.

Parameter	Name	Units	Levels	
			Low	High
**A**	Concentration of PLGA	%w/v	10	20
**B**	Solvent type	-	DCM	EAc
**C**	MW of PVA	Da	Low	High
**D**	Concentration of PVA in primary emulsion	%w/v	0.3	1.2
**E**	Vortexing speed	scale	5	8
**F**	Vortexing duration	seconds	45	90
**G**	Organic/aqueous phase ratio	v/v	1:1	1:3
**H**	Concentration of PVA in hardening bath	%w/v	0.3	1.2
**J**	Stirring speed	rpm	400	900
**K**	Solvent evaporation duration	hours	18	24

#### Taguchi OA design of experiments

Two types of Taguchi OA designs were implemented to develop a model for optimal PLGA MP formulation to produce a particle size in the range 10–50 μm. Taguchi design was employed to study only the main parameter effect. Taguchi L_12_ (2^10^) OA design (12, 2 and 10 represent the number of experiments, levels, and parameters respectively) was initially employed to study ten parameters at two levels: low and high (Table 1). The levels of the parameters were selected based on an established protocol by Bible *et al*. [16]. The results produced by the L_12_ OA design were fed into the second design: the parameters selection for Taguchi L_18_ (2^1^) (3^7^) OA design (18 = the number of experiments, 2 and 3 = levels, and 1 and 7 = the parameters) were guided by the analyses of a half-normal plot, Pareto chart and response plot generated for the L_12_ OA design. In the L_18_ OA design, the number of parameters was reduced to 8 and the number of levels for 7 parameters were increased to 3: low, medium and high (Table 2). The L_12_ OA and L_18_ OA data generated by design-expert software version 10 (Stat-Ease, Inc., USA) and measured responses are presented in Tables 3 and 4 respectively. Each row in these tables represents a formulation run with the level of the parameters. The “X50” particle size value representing the median particle diameter was taken as the response. All runs were performed in triplicate.

**Table 2:** Parameters and associated levels used in L_18_ design.

Parameter	Name	Units	Levels		
			Low	Medium	High
**A**	MW of PVA	kDa	13	89	-
**B**	Concentration of PLGA	%w/v	15	20	30
**C**	Concentration of PVA in primary emulsion	%w/v	0.8	1.2	2.0
**D**	Organic/aqueous phase ratio	v/v	1:0.75	1:1	1:2
**E**	Vortexing speed	scale	3	5	9
**F**	Vortexing duration	seconds	60	90	120
**G**	Stirring speed	rpm	100	400	1200
**H**	Solvent evaporation duration	hours	20	24	48

**Table 3:** Combination of parameter levels of L_12_ OA design.

Run	A (%w/v)	B	C (Da)	D (%w/v)	E (scale)	F (sec)	G (v/v)	H (%w/v)	J (rpm)	K (h)	Mean ± SD (μm)	S/N ratio
**1**	10	DCM	low	0.3	5	45	1:1	0.3	400	18	200.37 ± 2.82	-3.84
**2**	10	DCM	low	0.3	5	90	1:3	1.2	900	24	100.7 ± 12.12	-3.34
**3**	20	EAc	low	1.2	5	90	1:1	0.3	400	24	54.39 ± 4.37	-2.89
**4**	20	EAc	high	0.3	5	45	1:1	1.2	900	18	162.12 ± 4.83	-3.68
**5**	20	DCM	high	1.2	5	45	1:3	1.2	400	24	104.63 ± 0.85	-3.37
**6**	10	EAc	low	1.2	8	45	1:3	1.2	400	18	97.27± 0.46	-3.31
**7**	20	DCM	low	1.2	8	90	1:1	1.2	900	18	60.30 ± 1.29	-2.97
**8**	20	DCM	high	0.3	8	90	1:3	0.3	400	18	102.16 ± 7.19	-3.35
**9**	10	EAc	high	1.2	5	90	1:3	0.3	900	18	97.03 ± 0.08	-3.31
**10**	20	EAc	low	0.3	8	45	1:3	0.3	900	24	61.99 ± 16.27	-2.99
**11**	10	DCM	high	1.2	8	45	1:1	0.3	900	24	105.07 ± 1.73	-3.37
**12**	10	EAc	high	0.3	8	90	1:1	1.2	400	24	95.04± 1.89	-3.30

Note. Low = M_w_: 13–23 kDa, High = M_w_: 146–186 kDa

**Table 4:** Combination of parameter levels of L_18_ OA design.

Run	A: A (kDa)	B: B (%w/v)	C:C (%w/v)	D: D (v/v)	E: E	F: F (sec)	G: G (rpm)	H:H (h)	Mean ± SD (μm)	S/N ratio
**1**	13	15	1.2	1:1	5	90	400	24	49.76 ± 2.65	-2.83
**2**	89	30	0.8	1:2	5	120	200	24	45.48 ± 2.41	-2.76
**3**	13	20	2.0	1:2	3	60	400	24	60.84 ± 4.22	-2.97
**4**	89	20	2.0	1:0.75	5	120	400	20	41.12 ± 1.50	-2.69
**5**	89	15	1.2	1:0.75	3	120	1200	24	73.13 ± 0.52	-3.11
**6**	13	30	1.2	1:2	5	60	1200	20	44.94 ± 1.20	-2.75
**7**	89	15	2.0	1:1	5	60	200	48	25.83 ± 0.65	-2.35
**8**	13	30	0.8	1:1	3	120	400	48	67.09 ± 4.67	-3.04
**9**	13	20	1.2	1:1	9	120	200	20	24.01 ± 1.27	-2.30
**10**	13	30	2.0	1:0.75	9	90	200	24	50.81 ± 3.15	-2.84
**11**	13	15	2.0	1:2	9	120	1200	48	28.10 ± 1.26	-2.41
**12**	89	20	1.2	1:2	3	90	200	48	60.25 ± 6.09	-2.97
**13**	89	30	1.2	1:0.75	9	60	400	48	55.38 ± 1.07	-2.91
**14**	13	15	0.8	1:0.75	3	60	200	20	*	*
**15**	89	15	0.8	1:2	9	90	400	20	47.46 ± 5.39	-2.79
**16**	89	20	0.8	1:1	9	60	1200	24	23.51 ± 0.81	-2.29
**17**	89	30	2.0	1:1	3	90	1200	20	*	*
**18**	13	20	0.8	1:0.75	5	90	1200	48	40.38 ± 7.43	-2.68

Note.

* represents no creation of MPs

### Preparation of PLGA microparticles

PLGA MPs were fabricated using a single o/w emulsion technique. For the L_12_ design, 12 formulation runs were applied to produce MPs of different sizes. Accordingly, 0.5 or 1.0 g of PLGA were weighed and dissolved in 5 ml of DCM or EAc in a glass vial and incubated overnight at 19 °C to form a PLGA solution. 3.0 or 12.0 g of either 13 or 146 kDa PVA were completely dissolved in 1 L deionized water heated at 90 °C using an IKA heated magnetic stirrer (RCT basic, UK) at 800 rpm, followed by filtration. A hardening bath was formed by transferring 200 ml of filtered 0.3 w/v or 1.2% w/v PVA solution into a 250 ml glass beaker with a magnetic stirrer bar. 5 or 15 ml of the filtered 0.3 or 1.2% w/v PVA solution was added to the 10 or 20% w/v PLGA solution and shaken with Vortex-Genie^®^ 2 at a scale of 5 or 8 for 45 or 90 s (Scientific Industries, lnc, NY, USA) to form an o/w emulsion. The o/w emulsion was then added slowly to the hardening bath and stirred at either 400 or 900 rpm for 18 or 24 h for complete evaporation of the organic solvent and hardening of the MPs. The MPs were harvested using Whatman-grade 1 filter paper under vacuum. MPs were then washed three times with distilled water before being transferred to glass vials for freeze drying (MechaTech Systems Ltd, Bristol, UK) for 24 h.

The PLGA MPs in the L_18_ design were fabricated with the same single o/w emulsion technique and 18 different formulation runs were used. PVA solution at different concentrations was prepared by completely dissolving 8.0, 12.0 or 20.0 g of either 13 or 89 kDa PVA in 1 L deionized water. In order to form the o/w emulsion, 0.75, 1.0 or 1.5 g of PLGA were dissolved in 5 ml of EAC and added to either 3.75, 5 or 10 ml of 0.8, 1.2 or 2.0% w/v PVA solution. The solution was emulsified for 60, 90 or 120 s at a scale of either 3, 5 or 9. The o/w emulsion was homogenized into the hardening bath consisting of 200 ml of 0.3% w/v PVA (MW 13 kDa). The emulsion was stirred at either 100, 400 or 1200 rpm for 20, 24 or 48 h to ensure complete organic solvent evaporation. The prepared MPs were collected, washed and dried in a freeze drier for 24 h.

### Protein-loaded microparticles

The model formulation produced by the initial Taguchi method was exploited in the fabrication of drug-loaded PLGA MPs. Bovine serum albumin (BSA) was used as the model protein drug and conducted to validate the optimal locations of the parameter levels and also to investigate the effect of incorporated drugs on the particle size distribution. Briefly, 20 mg BSA was dissolved in 5 ml of ethyl acetate in which 1 g of PLGA polymer was completely pre-dissolved. A primary emulsion of the BSA/PLGA/EAc and PVA solution was formed and vortexed to create the microparticles. Thereafter, the emulsion was added to a hardening bath for allow for complete evaporation (24 hours). The supernatant was collected before the filtering, washing and freeze-drying of the generated particles.

Encapsulation efficiency (EE%) and loading capacity (LC%) were evaluated, through the indirect method of determining the amount of BSA in the supernatant, using Micro-QuantiPro^™^ BCA Assay Kit (Sigma-Aldrich, Poole, Dorset, UK). Thereafter, a mass balance calculation was performed to determine the amount of BSA loaded into the microparticles. The EE% and LC% were determined by the equations below:

$$EE\%=\frac{\left(Weight\;of\;initial\;BSA\;added-Weight\;of\;free\;nonentrapped\;BSA\right)}{Weight\;of\;initial\;BSA\;added}*100$$

$$LC\%\;=\;\frac{Weight\;of\;BSA\;entrapped}{Weight\;of\;BSA-loaded\;PLGA\;MP}*100$$

### Release profile determination

The *in vitro* protein release was determined using a method described by Determan *et al*, [37] with slight modification. Briefly, 50 mg of BSA-loaded PLGA MP were suspended in 3 ml of phosphate buffer saline (PBS). The samples were placed in a water bath at 37 °C and continuously stirred at 100 rpm. Sample volumes of (3ml) were collected at different times within 14 days. Each time, fresh preheated PBS was reintroduced to maintain sink conditions. The samples were centrifuged and the concentration of the protein in each sample was determined using Micro-QuantiPro^™^ BCA Assay kit.

### Particle size/polydispersity analysis

The particle size distribution was measured by laser diffraction using a SymPatec HELOS equipped with a RODOS/ ASPIROS dry dispenser (Germany). All measurements were performed using the R5 (4.5 to 875 μm) and R3 (0.5 to 175 μm) lens for L_12_ and L_18_ designs respectively. The particles were dispersed under a 4-bar pressure. About 5 mg of each sample was placed in an ASPIROS glass vial. The particle size distribution (volume mean diameter, X10, X50, X90) were analyzed with WINDOX 5 software (SymPatec, Germany).

### Scanning electronic microscopy of MPs

Surface morphology, size, and shape of the MPs fabricated by formulation runs were obtained using a scanning electron microscope (JOEL JCM-5700, USA). The dried MP samples were deposited onto adhesive carbon tabs (Agar Scientific G3357N), which were pre-mounted onto aluminum stubs (Agar Scientific JEOL stubs G306). The samples were gold sputtered for 60 s to attain a thickness of approximately 30 nm (Quorum SC7620). The morphologies of the MP samples were analysed at magnifications of X100, and X500.

### Statistical analysis

The experimental design results were statistically analysed using Design-Expert software version 10.0.5.0 (Stat-ease- Inc., Minneapolis). Response and interaction plots were generated to examine the effect of parameter levels on the mean response (particle size). Half-normal plots and Pareto charts were generated to guide the selection of parameters for the final optimal model. The data were assessed by ANOVA combined with Fisher’s statistical test (F- test) to determine whether a chosen parameter had a significant effect on the desired value (p < 0.05). The S/N ratio formula below (i.e. smaller-the-better) was used to evaluate the response values. All data presented were expressed as mean and standard deviation (SD).

$$\frac{S}{N}ratio\;=\;-10*\text{log}\frac{\sum (Y^{2})}{n}$$

where Y is the mean and n is the number of experiments.

## Results

### Microparticle formulation: L_12_ OA design

The Taguchi design DOE approach was explored to identify the process parameters in the solvent evaporation technique with the most significant effects on PLGA MP size and to generate a predictive model. L_12_ OA design was used as the first optimization step in the DOE. The parameters and their levels for the L_12_ OA design (Table 1) were selected based on previous studies using the solvent evaporation method for MP fabrication [16, 34–36]. From the design, ten parameters at two levels were investigated, namely; concentration of PLGA (A), solvent type (B), MW of PVA (C), concentration of PVA in primary emulsion (D), vortexing speed (E), vortexing duration (F), organic/aqueous phase ratio (G), concentration of PVA in hardening bath (H), stirring speed of the hardening bath (J), and solvent evaporation duration (K), with particle size as a response. This design resulted in 12 formulation runs (36 runs in total; three replicate for each run). PLGA MPs were then successfully fabricated using the 10 parameters combinations. Table 3 shows the parameter levels combinations computed by design-expert software for L_12_ OA design and the median particle size measurement by laser diffraction using a SymPatec HELOS equipped with a RODOS/ ASPIROS dry dispenser (Germany). The rows represent the formulation runs and the column represents the parameters. All the level settings in each formulation run appeared an equal number of time: for each parameter, low level and high level appeared 6 times. The data with the average median particle size ranging from 54.39 to 200.37 μm was produced for the design. The data had a mean of 103.42 ± 41.55 μm. None of the runs produced MPs within the goal range of 10–50 μm. Nonetheless, Run 3 (54.39 μm) created MPs with smallest median particle size closed to the upper range value. MPs with smallest median diameter were obtained from an organic phase comprising of EAc and 20% w/v PLGA polymer, and an aqueous phase of low molecular weight of 1.2% w/v PVA which were combined at a ratio of 1:1 to form a primary emulsion by a vortexing speed at scale 5 for 90 s. The emulsion was introduced to a hardening bath of 0.3% w/v PVA at a speed off 400 rpm and the MPs created were hardened at complete solvent evaporation for 24 h (Run 3). The largest particle size was registered with 10% w/v PLGA concentration, DCM, 0.3% w/v (low MW) PVA in primary emulsion, vortexing speed at scale 5, vortexing duration of 45 s, 1:1 organic/aqueous phase ratio, 0.3% w/v PVA in hardening bath, 400 rpm stirring speed and 18 h evaporation duration (Run 1).

#### Statistical data analysis (L_12_ OA design)

**Fig 1:**
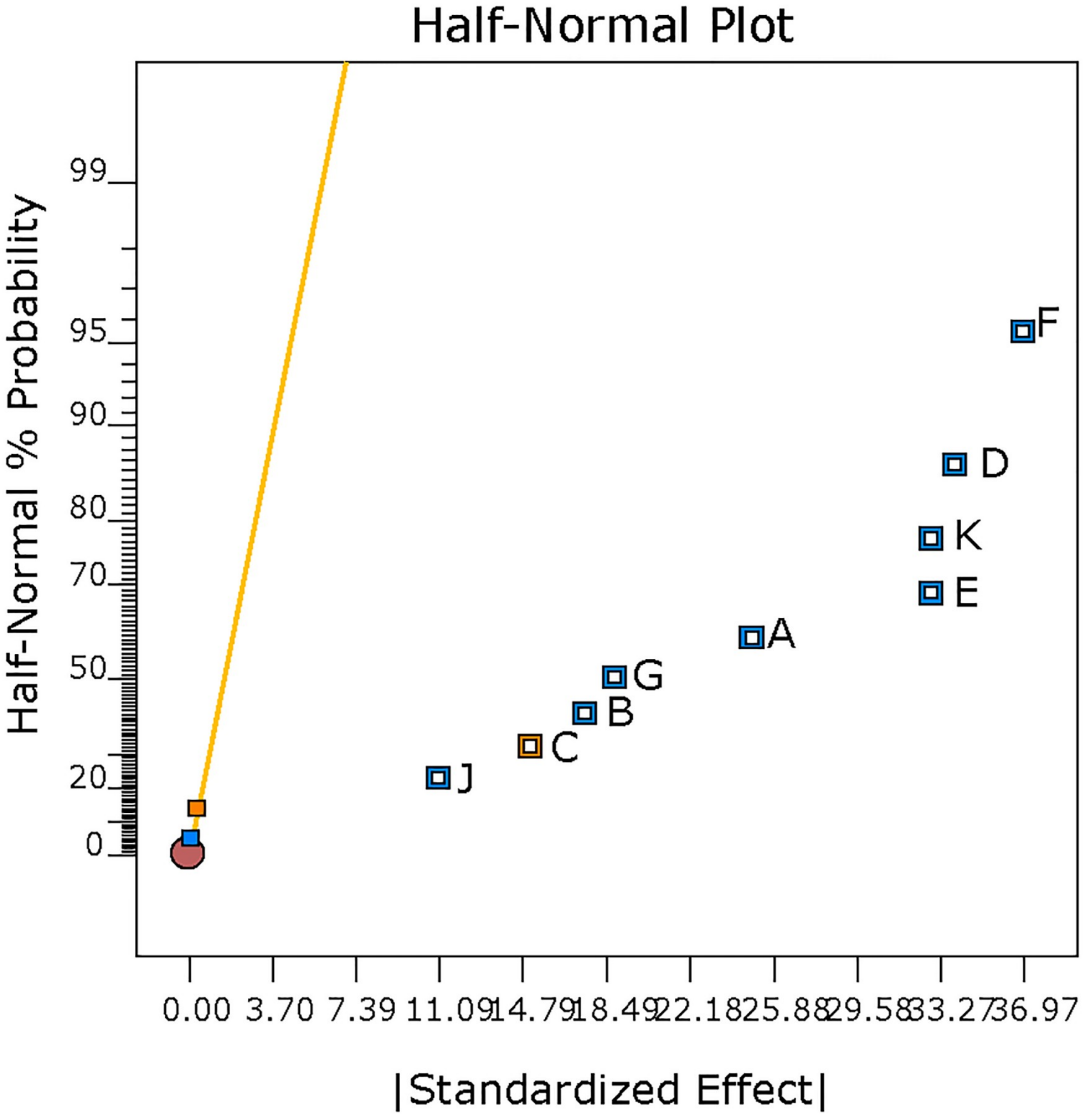
Half-normal % probability versus standardized effects plot after selection of parameters for the model. The yellow line represents the error line. Positive effects represent parameters with positive standardized values and negative effects signify parameters with negative standardized values. Plot generated for L_12_ OA design parameters: stirring speed (J), molecular weight of PVA (C), solvent type (B), organic/aqueous phase ratio (G), concentration of PLGA (A), vortexing speed (E), solvent evaporation duration (K), concentration of PVA in emulsion (D), vortexing duration (F).

**Fig 2:**
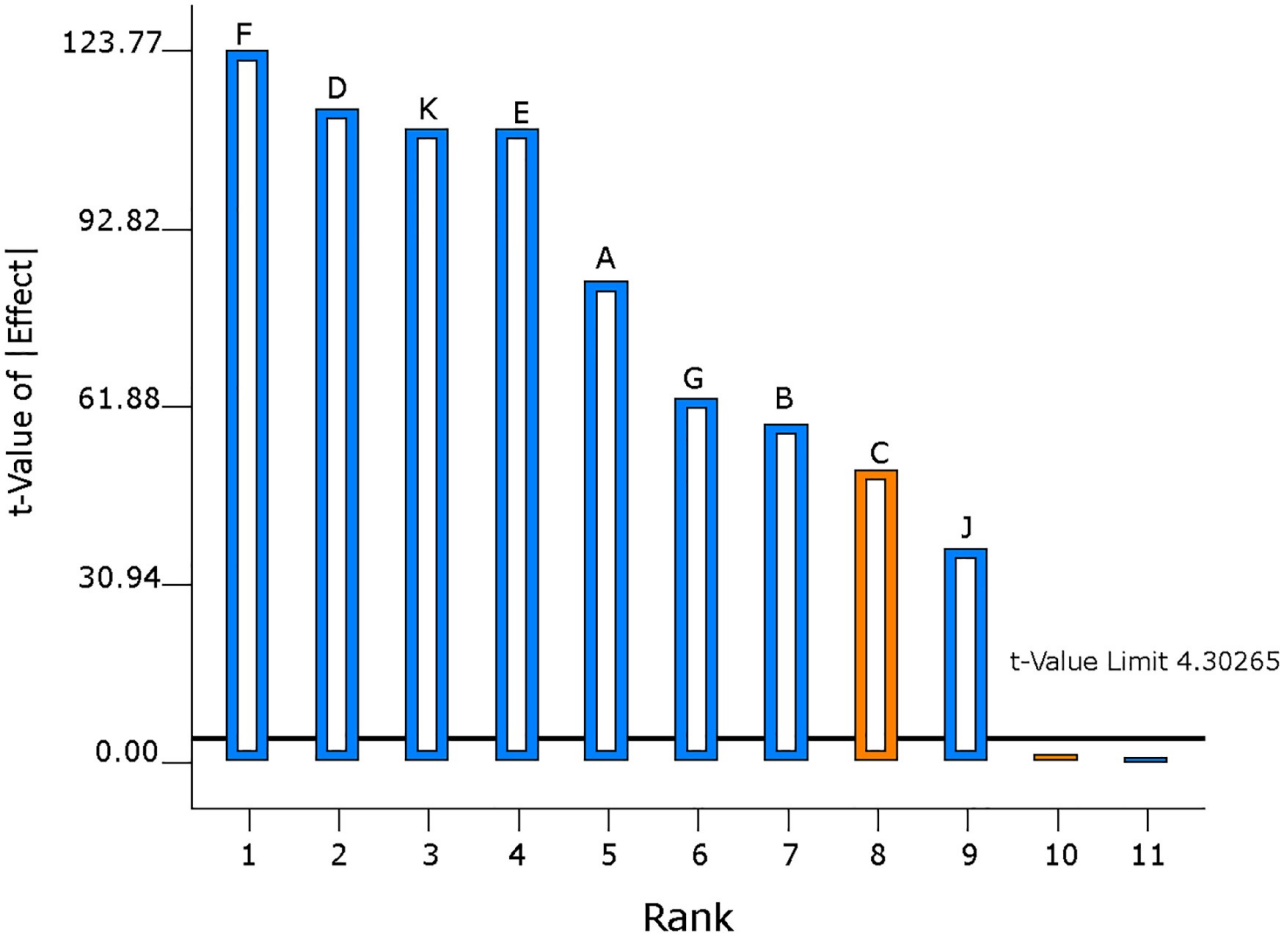
Graph of t-value of absolute effects verses rank. This is an ordered bar chart, which shows the magnitude of the chosen parameters for the model. The bars above the t-value (the reference line) with white represent the significant effects and the bars below the reference line are insignificant. Data generated for L_12_ OA design parameters: vortexing duration (F), concentration of PVA in emulsion (D), solvent evaporation duration (K), vortexing speed (E), concentration of PLGA (A), organic/aqueous phase ratio(G), solvent type (B), molecular weight of PVA (C) and stirring speed for hardening process (J).

**Fig 3:**
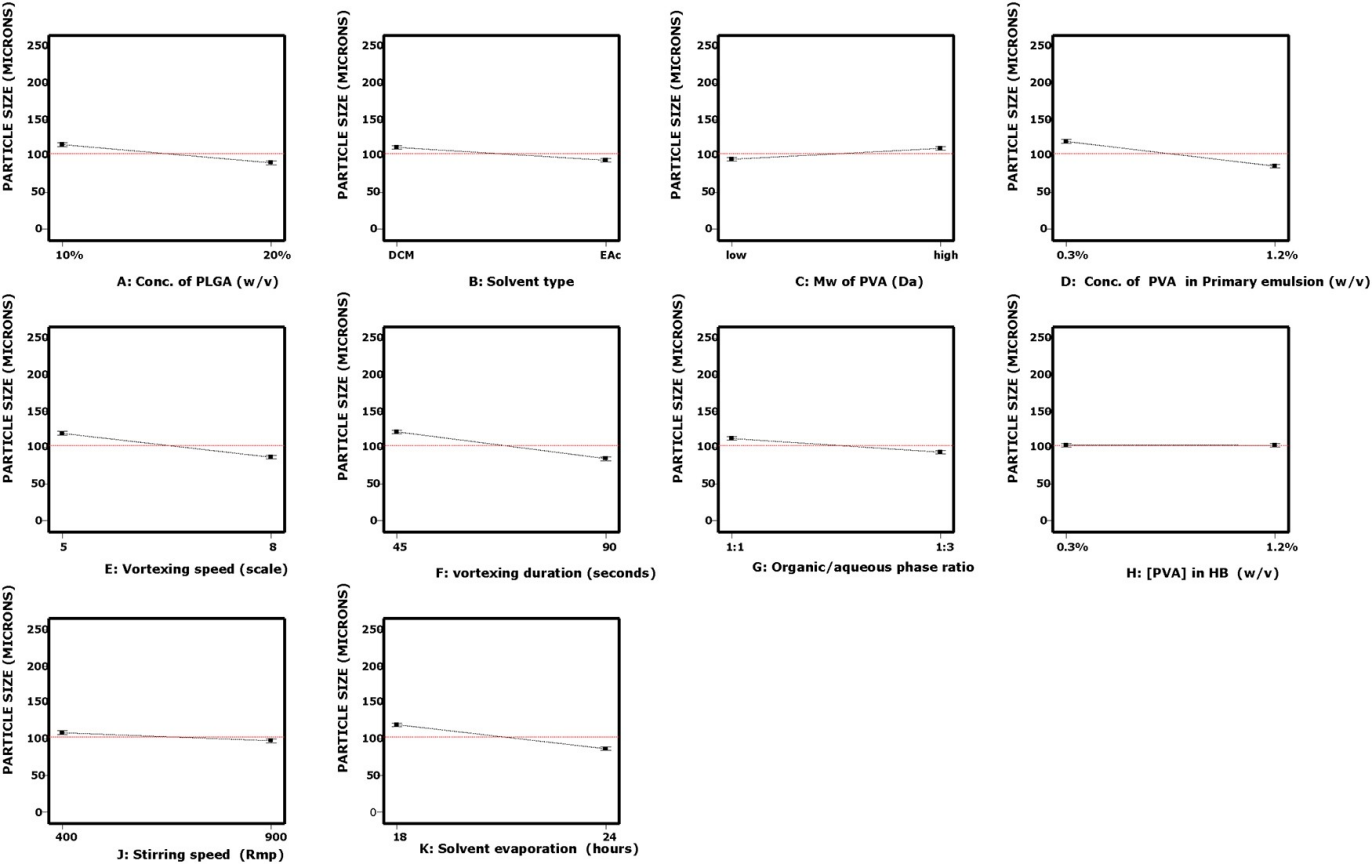
Response (main effects) plots for the average effects of the 10 process parameters (A, B, C, D, E, F, G, H, J and K) on the mean particle size for the L_12_ OA design. The vertical axis shows the mean particle size (μm) and the horizontal axis shows two levels (low and high) of the process parameters. The red dashed line represents the value of the total mean of the particle size.

**Fig 4:**
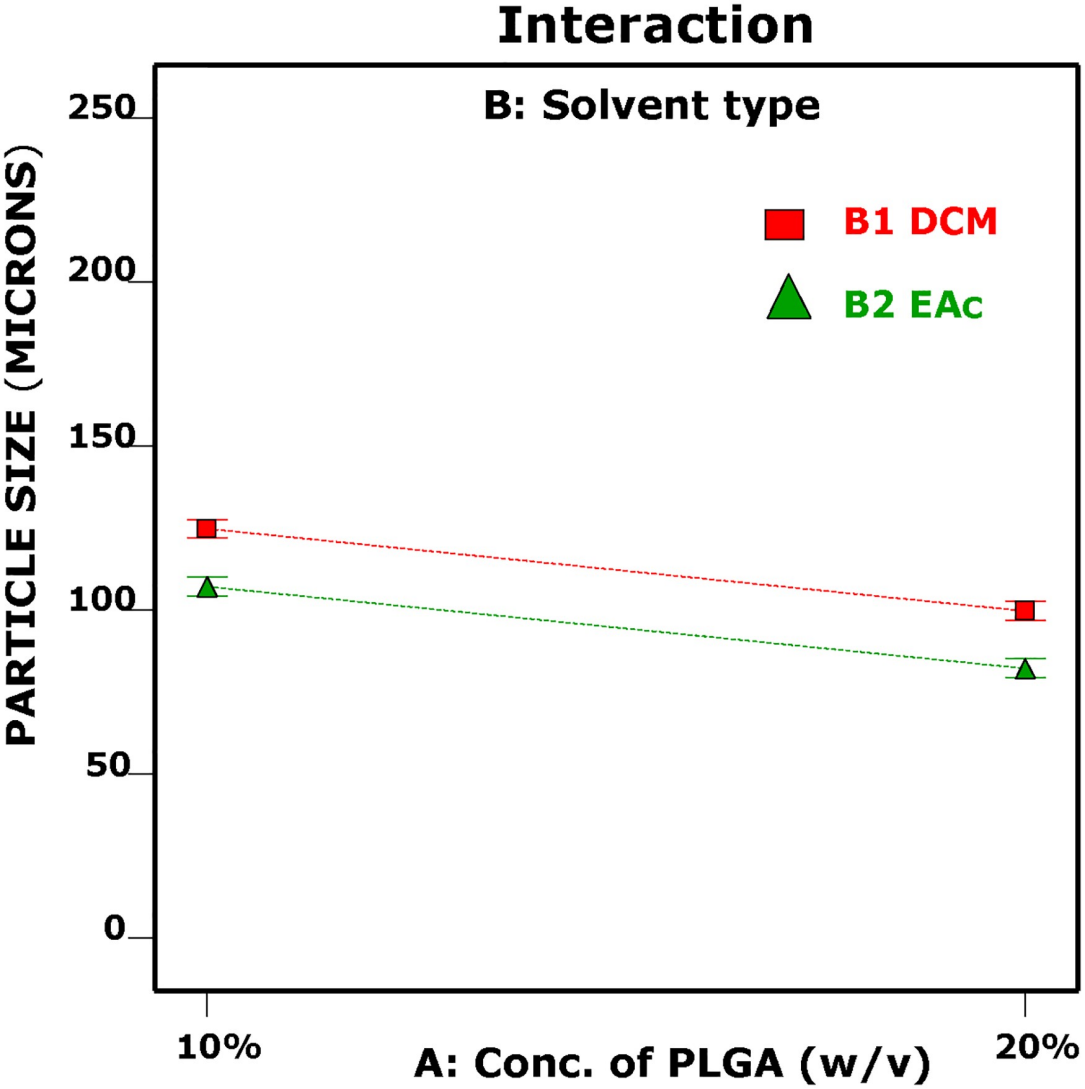
Interaction graph plot showing the effect of concentration of PLGA (w/v) and solvent type on the mean particle size (μm).

S/N ratio was computed for each of the formulation run with the smaller-the-better particle size characteristic (Table 3). High S/N ratio value was recorded for formulation Run 3 (-2.89). A half-normal probability plot was generated by using the estimated effects of the parameters to help assess the important and unimportant parameters (Fig 1). The following parameters: vortexing duration, concentration of PVA in emulsion, solvent evaporation duration, vortexing speed, concentration of PLGA, organic/aqueous phase ratio, solvent type, molecular weight of PVA and stirring speed are found at the right side of the plot and parameters: concentration of PVA in hardening bath and interaction between concentration of PLGA and solvent type lined up on the yellow error line. A Pareto chart was generated to identify the magnitude of the chosen effects for the model formulation (Fig 2). It was observed that all bars of the parameters are above the t-test (reference line) except the concentration of PVA in the hardening bath and interaction between the concentration of PLGA and solvent type. The white column seen inside the bars indicates that the parameters have significant effect on the mean particle size. Table 5 displays the ANOVA results for the particle size of the MP to study the significant parameters included in the model. F-test was carried out on the experimental data and a value of 7880.15 was obtained. All the parameters chosen for the model have significant effects on the MP size. From Table 5, vortexing speed had the maximum contribution to the total variance (21.60%) and the least was stirring speed for the hardening process (1.95%). Additionally, a diagnostic analysis was performed by generating the response plots for the 10 parameters to evaluate the influence of each average parameters levels on the mean particle size (103.4 μm) (Fig 3). The plot for levels of the concentration of PVA in hardening bath shows a negligible effect on the particle size. However, the plots for the remaining parameters show the high levels affecting the particle size significantly except the MW of PVA with the low level having an influence on the particle size. Fig 4 is the interaction plot for parameter A and B displaying parallel lines to indicate no influence on the mean particle size. The results obtained in the plots (Figs 3 and 4) are the same as the results presented in the ANOVA table.

**Table 5:** ANOVA for L_12_ OA design.

Source	Sum of Squares	Degree of freedom (df)	Mean Square	F Value	p-value Prob. > F	% Contribution
**Model**	18986.23	9	2109.58	7880.15	0.0001	-
**A**	1872.25	1	1872.25	6993.62	0.0001	9.86
**B**	925.59	1	925.59	3457.45	0.0003	4.87
**C**	690.54	1	690.54	2579.44	0.0004	3.64
**D**	3457.47	1	3457.47	12915.06	< 0.0001	18.21
**E**	3247.56	1	3247.56	12130.96	< 0.0001	17.10
**F**	4100.71	1	4100.71	15317.84	< 0.0001	21.60
**G**	1073.71	1	1073.71	4010.75	0.0002	5.66
**J**	370.19	1	370.19	1382.79	0.0007	1.95
**K**	3248.22	1	3248.22	12133.42	< 0.0001	17.11
**Residual**	0.5354	2	0.2677	-	-	-
**Corr. Total**	18986.76	11	-	-	-	-

### Microparticle formulation: L_18_ OA design

**Fig 5:**
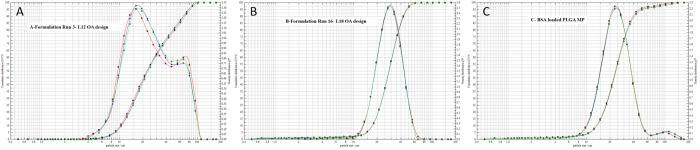
Cumulative size distribution of PLGA MP fabricated with ethyl acetate as organic solvent: (A) formulation Run 3 from L_12_ OA design and (B) formulation Run 16 from L_18_ OA design identified as the optimal model formulation (n = 3).

**Fig 6:**
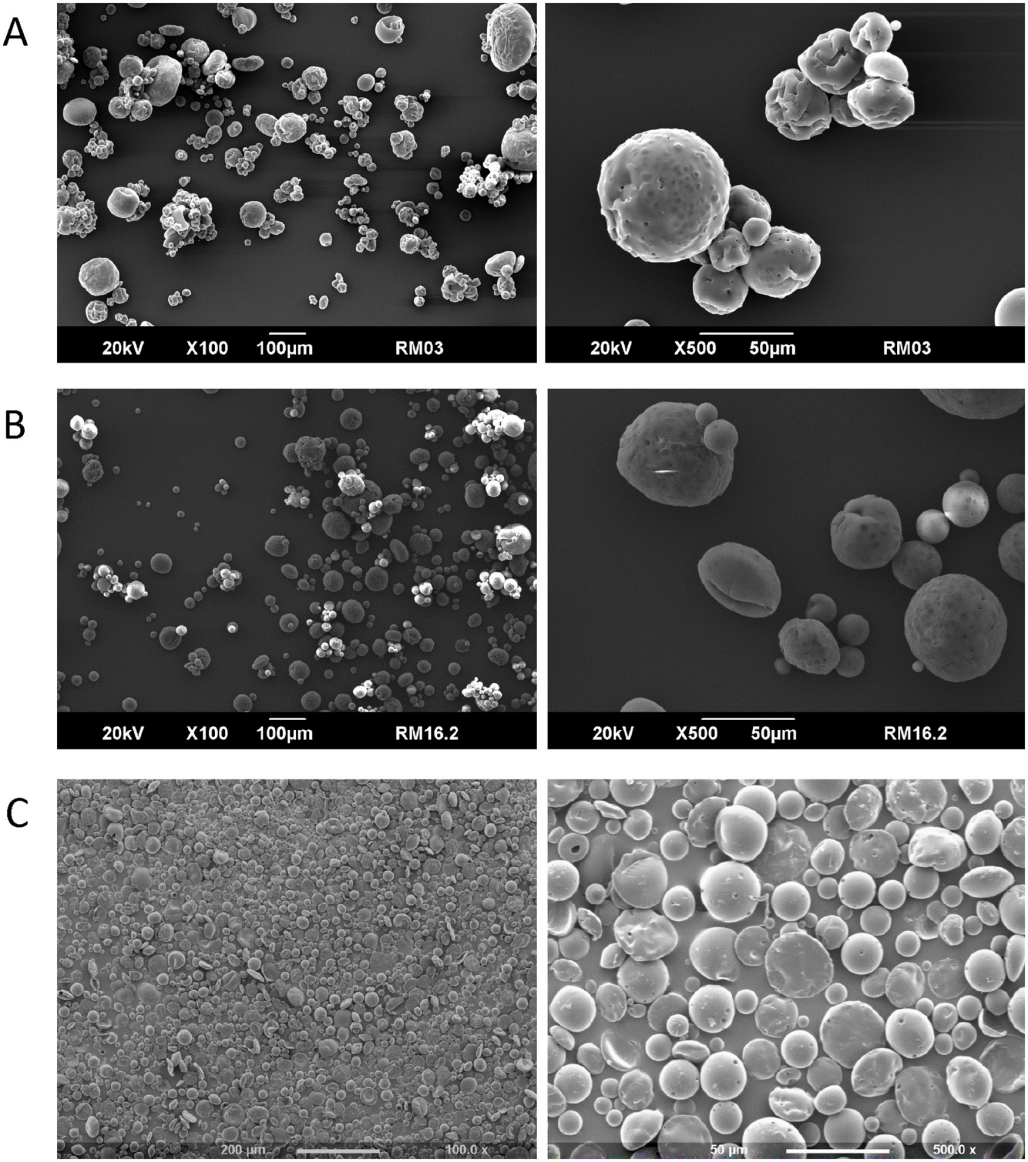
SEM images of PLGA MP formulated by (A) run 3 from L_12_ OA design and (B) run 16 from L_18_ OA design (optimal model formulation) and (C) BSA-loaded PLGA MP.

The L_12_ OA design identified 9 important parameters: concentration of PLGA, solvent type, MW of PVA, concentration of PVA in primary emulsion, vortexing speed, vortexing duration, organic/aqueous phase ratio, stirring speed of the hardening process and solvent evaporation duration that significantly influence the particle size of MP as such can be controlled. These results were fed into a second design, L_18_ OA design to further ascertain how these parameters determine the particle size of MP by introducing a third level for each of the important parameters excluding parameter C (i.e. solvent type). Eight parameters: MW of PVA (A), concentration of PLGA (B), concentration of PVA in primary emulsion (C), organic/aqueous phase ratio (D), vortexing speed (E), vortexing duration (F), stirring speed of the hardening process (G) and solvent evaporation duration (H) were considered for the L_18_ OA design. The parameter levels as seen in Table 2 were based on formulation Run 3 as this generated the smallest particle size closed to the target range. The solvent type (EAc and PVA) concentration in the hardening process (0.3% w/v) were kept constant for all the formulation runs. The particle sizes were measured by laser diffraction technique. Table 4 shows the L_18_ design generated by the design-expert software and the particle size obtained for each formulation run. A data of 18 formulation runs with 8 parameters was constructed, in each of the parameters column, the levels low, medium or high occurred 9 times. The 16 formulation runs within this design produced particle size range of 23.51–73.13 μm and a mean particle size of 48.32 μm. Runs 14 and 17 failed completely and no MPs were created. These formulation runs failed using scale 3 as the vortexing speed to homogenize the o/w emulsion. Looking at Table 4 most of the MP created from this design have particle size within the target range apart from Run 3 (60.84 μm), Run 5 (73.13 μm), Run 8 (67.04 μm), Run 10 (60.25 μm) and Run 12 (55.38 μm). MPs formulated with Runs 3, 5, 8 and 10 had large particle size by using vortexing speed at the low level (scale 3). The goal of the study was to generate the smallest particle size within the range 10–50 μm, the smallest particle size 23.51 μm (PDI = 1.09 ± 0.01) was achieved by formulation Run 16 and the cumulative size distribution curves for this run are shown in Fig 5. SEM images were taken to visualise the surface morphology of the MP formulation Run 16 selected from L_18_ OA design as the optimal model formulation for this study. The SEM images of the MP samples are shown in Fig 6. The images shows that each sample are made of a range of spherical microparticles and these confirm the particle size measurements. The data for the SEM and the size distributions were compared to the formulation Run 3 in L_12_ OA design.

#### Statistical data analysis (L_18_ OA design)

**Fig 7:**
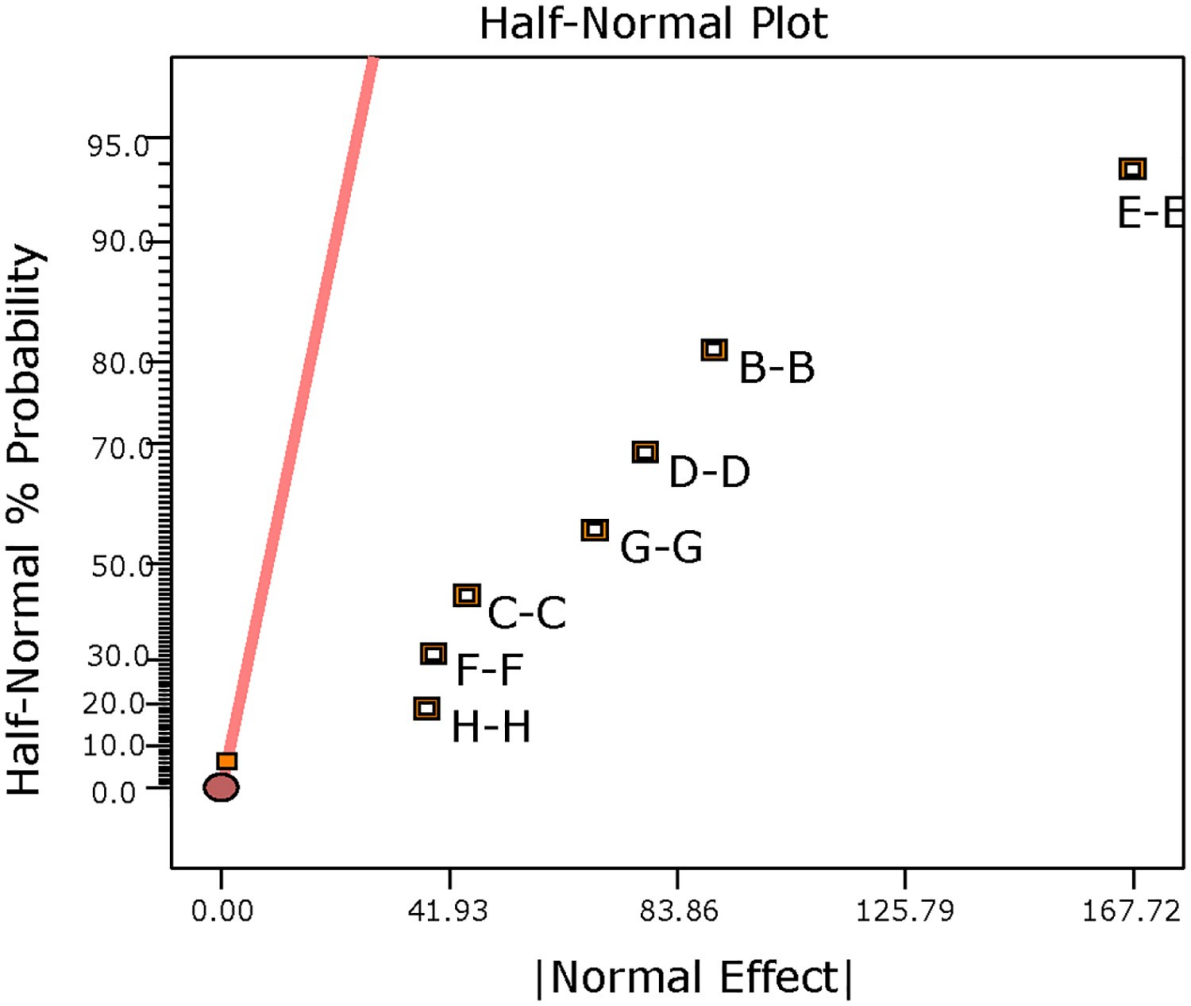
Half-normal % probability versus normal effects plot after selection of parameters for the model. Pink line represents error line. Data generated for L_18_ OA design parameters: vortexing speed (E), concentration of PLGA (B), organic/aqueous phase ratio (D), stirring speed (G), concentration of PVA in emulsion (C), vortexing duration (F) and solvent evaporation duration (H).

**Fig 8:**
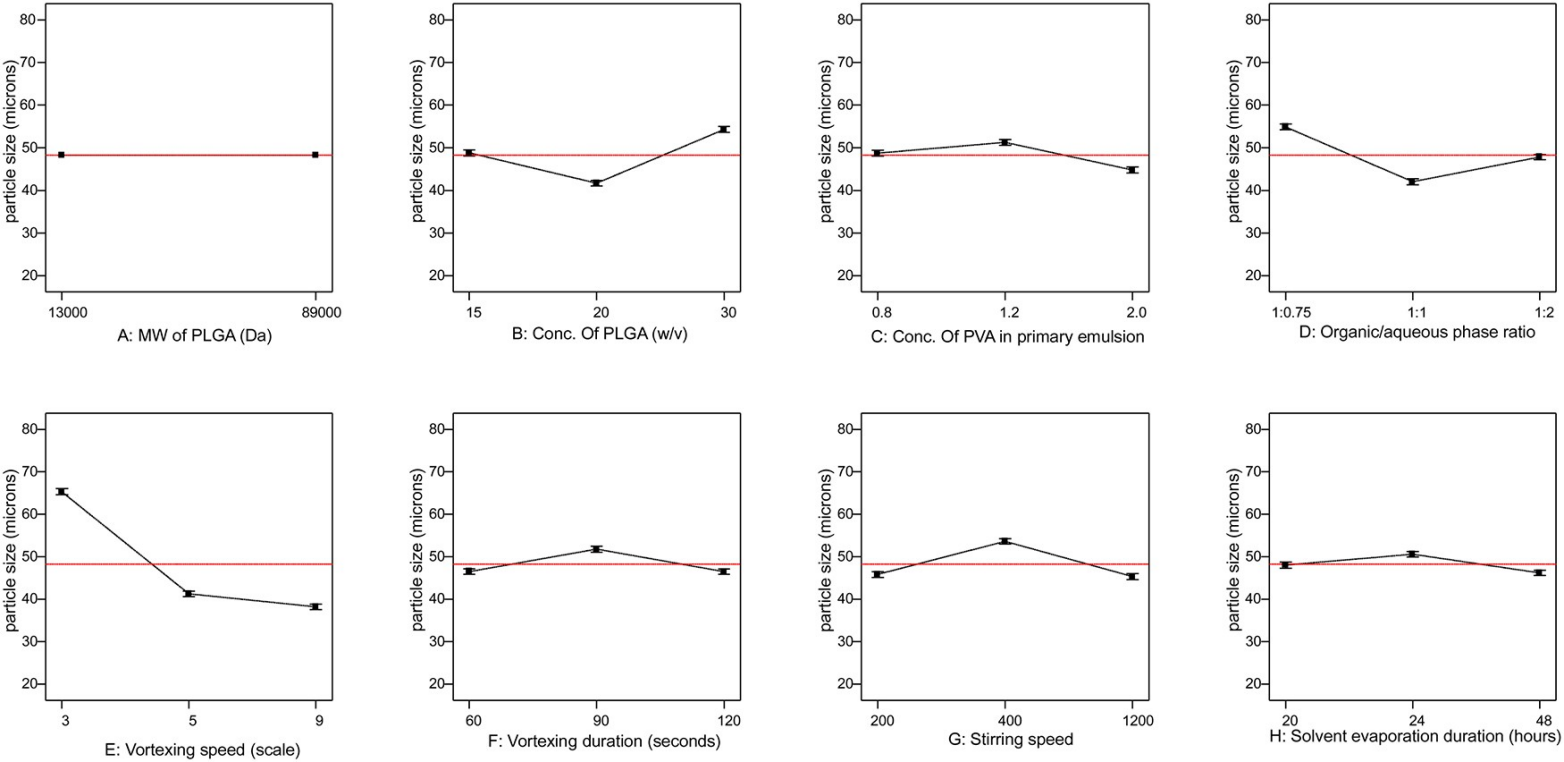
Response plots for the average effects of the 8 process parameters (A, B, C, D, E, F, G and H) on the mean particle size for the L_18_ OA design. The vertical axis shows the mean particle size (μm) and the horizontal axis shows three levels (low, medium and high) of the process parameters. The red dashed line represents the value of the total mean of the particle size.

Table 4 summarises the S/N ratio evaluated for each of the formulation runs. The largest value based on the smaller-the-better analysis was produced by Run 16 (-2.29). Fig 7 displays the half-normal plot generated by the design-expert software to assess which of the parameters are important and which are unimportant. The parameters: vortexing speed, concentration of PLGA, organic/aqueous phase ratio, stirring speed, concentration of PVA in primary emulsion, vortexing duration and solvent evaporation duration are seen at the far right of the error line. The parameter MW of PVA is located on the error line. The ANOVA for the selected parameters: concentration of PLGA, concentration of PVA in primary emulsion, organic/aqueous phase ratio, vortexing speed, vortexing duration, stirring speed of the hardening process and solvent evaporation duration is summarized in Table 6 and it shows all parameters selected for the model have significant effects on the particle size (p >0.05). A high value of F (12261.0) was obtained for the model (1.68%). The sum of squares shows that 32.79% of the total variance is established by the vortexing speed. The parameter with the least contribution is solvent evaporation duration. Fig 8 shows the response plots for the average parameter levels of the L_18_ design. No influence is observed for the two levels of the MW of PVA. The concentration of PLGA response shows medium level (20% w/v) having the largest influence on the mean particle size followed by the low level (15% w/v) and then the high level (30% w/v). The plot for the concentration of PVA in primary emulsions displays 2.0% w/v (high level) with the most significant effect and the 1.2% w/v (medium level) with the least. The medium level (1:1) for the organic/aqueous phase ratio increased the mean particle size while the high level (1:3) and low level (1:0.75) decreased the response accordingly. As the level increases in the vortexing speed parameter, the effect on the mean particle size increases. In the plot for vortexing duration, the low level (60 seconds) and the high level (120 seconds) have an equal and larger influence on the mean particle size. Parameters: stirring speed and solvent evaporation duration have the same pattern for levels influence on the mean particle size i.e. low > high > medium implies a reduction in the effect.

**Table 6:** ANOVA for L_18_ OA design.

Source	Sum of Squares	df	Mean Square	F Value	p-value Prob. > F	% Contribution
**Model**	3504.60	14	250.33	12261.00	0.0071	
**B-B**	336.50	2	168.25	8240.73	0.0078	9.60
**C-C**	83.86	2	41.93	2053.66	0.0156	2.39
**D-D**	249.47	2	124.74	6109.56	0.0090	7.12
**E-E**	1149.15	2	574.58	28142.50	0.0042	32.79
**F-F**	62.67	2	31.34	1534.89	0.0180	1.79
**G-G**	193.63	2	96.82	4742.02	0.0103	5.53
**H-H**	58.75	2	29.38	1438.86	0.0186	1.68
**Residual**	0.0204	1	0.0204	-	-	-
**Corr. Total**	3504.62	15	-	-	-	-

### Characterization of BSA-loaded PLGA MP

**Fig 9:**
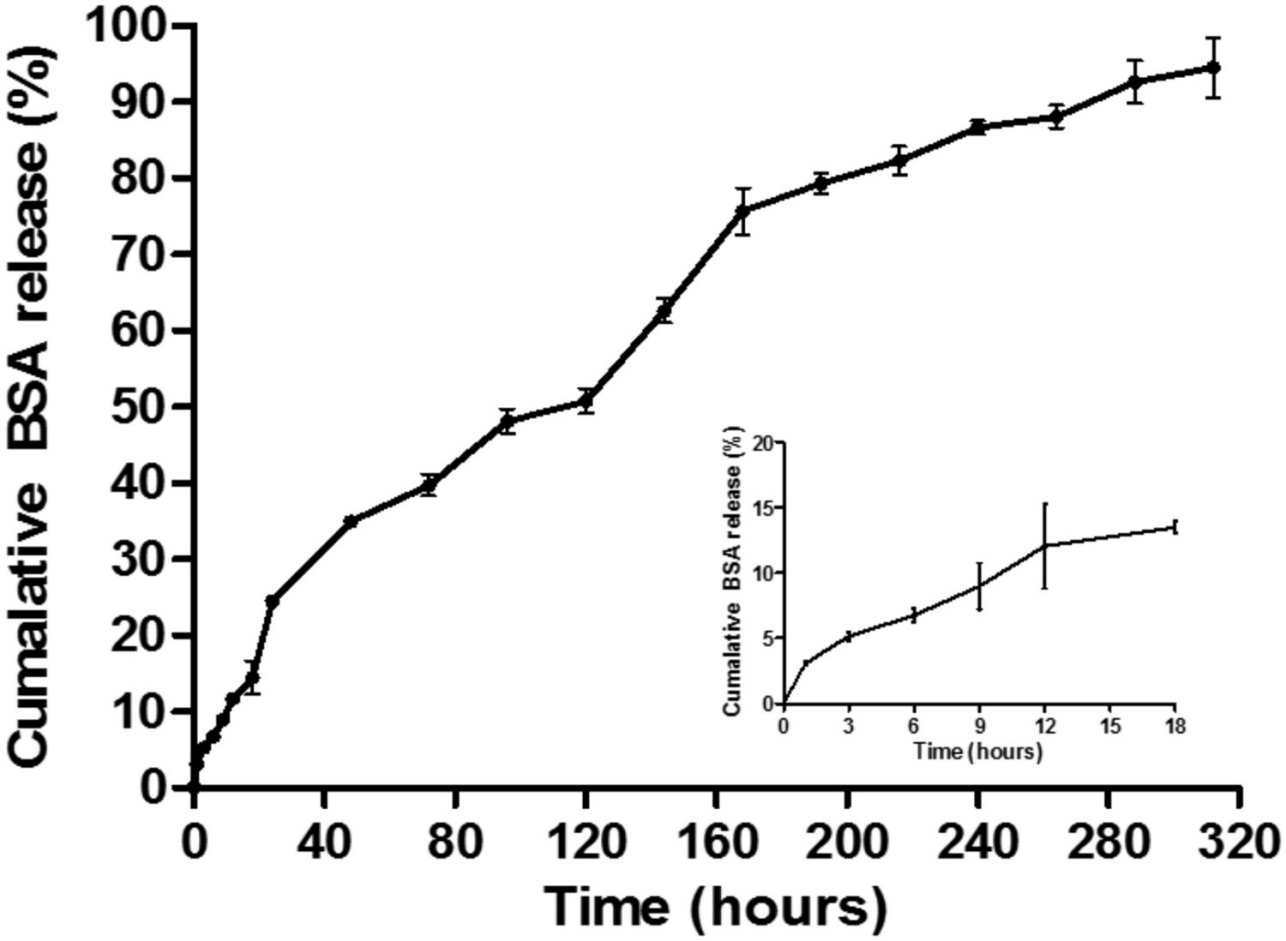
The *in vitro* release of BSA from MP prepared with the optimal model formulation. Results are described as mean± SD, n = 3.

BSA-loaded PLGA MPs were successfully generated using the parameters derived from the optimal model formulation. The average particle size was determined as 22.79 ± 0.08 with a PDI of 1.13 ± 0.04, which is not significantly different than the unloaded microparticles (p > 0.05 by t-test). The average EE% and LC% (of the BSA protein) in these MPs were quantified as 56.37 ± 1.26 and 15.03 ± 0.89, respectively. In addition, SEM analysis of the BSA-loaded PLGA MP further confirmed the results obtained from the particle size analysis and also documented the spherical shape of these MPs (Fig 6). The *in vitro* release profile of the incorporated protein from the BSA-loaded PLGA MP is summarized in Fig 9 where it can be seen that a steady-rate of release is maintained over a period of 13 days, at which >90% of total BSA is released from the MPs.

## Discussion

The solvent evaporation method has been used widely to encapsulate drugs in polymer matrices to form delivery systems and involves many processing parameters which influence the properties and quality of the final MPs product [12–16]. Optimising this process one variable at a time is impractical. As such, this study employed the use of a Taguchi orthogonal arrays design of experiment protocol to assess the optimal conditions required for the fabrication of 10–50 μm sized MPs and to identify the parameters that deemed to most significantly influence PLGA MP size.

The first optimization step in this study was the implementation of L_12_ OA design to evaluate 10 parameters at two levels (low and high) and identify the parameters having influence on particle size. This design is meant for evaluating the effect of 10 independent parameters, each having two level values. This design assumes that there is no interrelatioship between any two parameters. However, a clear evidence of interaction was observed between concentration of PLGA and solvent type, although this interaction had no effect on the particle size. This is because in each formulation run the PLGA polymer was dissolved in a constant volume (5 ml) of solvent type stated in the run. The mean particle sizes (response) for the 12 experiments were in a range of 54.39–200.37 μm as shown in Table 3. MPs with the largest particle size (200.37 μm) were formulated with Run 1 when all the 10 parameters had been used in their lowest levels. The smallest mean particle size (54.39 ± 4.37 μm) was obtained from Run 3 in which the concentration of PLGA, solvent type, concentration of PVA primary emulsion, vortexing duration, and solvent evaporation duration were ‘high’ and the molecular weight of PVA, vortexing speed, organic/aqueous phase ratio, concentration of PVA in hardening bath and stirring speed during the hardening process were at their low level. In this experiment, a smaller-the–better response (particle size) was desirable and the S/N ratio was calculated for each of the 12 experiments run in order to evaluate the data and identify the best set of parameters levels to generate the smallest particle size. Form the results, formulation Run 3 produced the smallest particle size (54.39 μm) with the highest S/N ratio value (-2.89).

The L_12_ OA design results were statistically analysed using design-expert software (version 10). This software is able to screen each parameter with respect to their influence on the particle size characteristic. A half-normal plot is one of the tools used in this software by identifying the important parameters and any interactions. This plot uses the magnitude of the estimated effects for the main parameters and any interactions in order to evaluate the important and unimportant parameters, and order the parameters from the most important down to the least important. Important parameters have their estimated effects completely removed from zero, while unimportant parameters have near-zero effects [38]. From Fig 1, the parameters located at the far right of the error lines are the important parameters and parameter F(vortexing speed) can be considered to have the maximum independent effect on particle size. Concentration of PVA in hardening bath and the interaction between the concentration of PLGA and solvent types that are lined up on the error line are unimportant, have zero or near zero effect on the particle size and excluded from the model. In Fig 1, the orange colour represents the parameter that has a positive effect and the blue represents parameter with negative effect on the mean particle size. In addition, a Pareto chart was created to identify the magnitude of the chosen parameters effects for the model (Fig 2). The bars above the t- critical value (the reference line) with white represent the important parameters that can possibly be included in the model and the bars below the reference line are unimportant. The white column seen inside the bars indicates that the parameters have significant effect on the mean particle size. F-test was carried out on the experimental data. The ANOVA for the selected model summarised in Table 5 showed that the model F value of 7880.15 was significant and that there was only 0.01% likelihood the variations among the mean particle size of the parameters is due to noise. Furthermore, a p-value of less than 0.05 indicates that the model terms are significant at the probability level of 95%. All the parameters chosen for the model have very significant effects on the microparticle size. From Table 5, vortexing speed had the maximum contribution (21.60%) and the least was stirring speed for the hardening process (1.95%). This evaluation is confirmed in the data presented in the Pareto chart (Fig 2).

The effect of each parameter on the mean paricle size are clearly observed in the response plots (Fig 3). The plots illustrate the average of each particle size for each level of each parameter and display the parameter with the largest effect. From the plots, the concentration of PVA in hardening bath (H) has negligible effect on the mean particle size. However, the remaining parameters show significant effects on the mean particle size as already evaluated by AVOVA and the Pareto chart. As shown in Fig 3, increasing the vortexing duration (F) produced a small particle size. An extended duration of vortexing may allow for better dispersal of the oil phase to form fine droplets which then harden to form MPs [39, 40]. The effective creation of a primary emulsion and the stability of the droplets during the emulsification depends on the presence of surfactant, in this case PVA, at the interface between the aqueous and organic phases, lowering interfacial tension and providing a barrier to coalescence [35,36,40]. Increasing the PVA concentration in the primary emulsion (D) from 0.3% w/v to 1.2% w/v resulted in a decrease in the mean particle size (Fig 3). As shown in the figure, an increase in the solvent evaporation duration (K) resulted in a decrease in mean particle size. It is possible that at the low level solvent removal from the droplet is incomplete, and that a longer duration allows for removal of any residual solvent expanding the polymer network. Vortexing speed (E) was a parameter of primary importance in the homogenization step because energy is required to disperse the organic phase in the aqueous phase [39]. The results showed that the mean particle size was inversely proportional to the vortexing speed; increase in vortexing speed decreased the MP size because the emulsion was dispersed into smaller droplets at a higher scale. This observation is line with the studies of Sharma, Madan and Lin [36]. The response plot for PLGA concentration (A) in the figure clearly shows that particle size decreased at higher PLGA concentration and may be as a consequence of PLGA surface activity. In this study two organic/aqueous phase ratios (G) were evaluated (1:1 and 1:3). The higher continuous phase volume may allow for a greater distance between dispersed oil droplets, reducing the rate of collision and coalescence [41, 42]. As observed in Fig 3, microparticles prepared with solvent type (B), EAc were characteretized by their smaller particle size and most likely due to the result of increased water solubility. The response plot for MW of PVA (C) shows that increasing the molecular weight increases the microparticle size and may be attributed to the increased viscosity of the solution reducing shear during emulsification [43, 44]. Stirring speed is one of the parameters that is well documented to have significant effect on particle size [16, 36, 37, 45–49]. Increasing stirring speed produces microparticle with small sizes by improved dispersal of the oil phase. This observation is confirmed in this study. In the effect of stirring speed (J) plot, the particle size decreased with increased in stirring speed from 400 rpm to 900 rpm.

From the analysis of the L_12_ OA design, 9 parameters were identified as important to be included in the formulation model but 8 parameters were used for the L_18_ OA design: this “exclusion” was based on the fact that the solvent used i.e. ethyl acetate is less polar than DCM and so there was no justification for considering a more polar solvent for further analyses. As such, the L_18_ OA design parameters included: concentration of PLGA (B), concentration of primary emulsion (C), organic/aqueous phase ratio (D), vortexing speed (E), vortexing duration (F), stirring speed (G) and solvent evaporation duration (H) at three levels: low, medium and high and 1 parameter: molecular weight of PVA (A) at two levels: low and high (Table 2). The parameters levels ranges were selected based on the levels of the Run 3 formulation in L_12_ OA design. Table 4 represents the L_18_ OA generated by design- expert software and the mean particle size measured for each of the 18 experimental runs. The mean particle size range obtained was 23.51 to 73.13 μm. From the Fig 5, the generated half normal plot shows, vortexing speed, concentration of PLGA, organic/aqueous phase ratio, stirring speed, concentration of PVA in emulsion, vortexing duration and solvent evaporation duration as the important parameters for the model. In the figure, the orange colour indicates the parameters have positive effect on the mean particles size. ANOVA has shown that the parameters have a significant effect (Model F value = 12261.0, p < 0.05) on the mean particle size (Table 6). There is only 0.71% likelihood that this large model F value could occur due to noise. Except for the MW of PVA, all other parameters do significantly influence the mean particle size. From these analyses, the major paramenter affecting the particle size is vortexing speed (E) with 32.79% and the least ranking parameter is solvent evaporation duration, 1.68% (H).

The influence of each parameter on the mean particle size are clearly observed in the response plots (Fig 7). The effect of MW of PVA (A) on the particle size is negligible. The results show that increasing the vortexing speed (E) from scale 3 to scale 9 reduced the particle size. Changing the concentration of PLGA (B) and the organic/aqueous phase ratio (D) from a low level of to meduim level decreased the particle size. However, an increase to high level caused an increasing effect on the particle size. The low and high levels of parameters: stirring speed (G), PVA concentration in primary emulsion (C), vortexing duration F) and solvent evaporation duration reduce the particle size. Whereas the medium levels have increased the particle size. Crucially, the shape of the fabricated (spherical) MPs do not change with the different parameters/within each Run. Microparticulate formulations are often considered in respect to the development of a drug delivery system for clinical applications. Herein, we additionally assessed the robustness and predictive ability of the Taguchi OA design of experiment methodology in the fabrications of the BSA-loaded MPs. BSA, ~66KDa, is a stable, globular and relatively non-reactive protein that is often used as a model protein in drug MP development studies [50]. As such, data presented in this study demonstrate that following sufficient entrapment of BSA within the MPs (i.e. >56% encapsulation efficiency), no significant changes in size occur- in agreement with the Taguchi L_18_ OA assessment. This aspect further validates the DOE technique as a feasible predictive tool for pharmaceutical formulation- especially in the context of biological entrapment e.g. growth factors, antibodies, biologicals.

Taken together, these results highlight the complexity of the MP fabrication process and the clear benefits of a DOE approach. The optimal model formulation was identified to be Run 16 from the L_18_ OA design. The mean particle size was found to be 23.51 μm which falls ino the required range of 10–50 μm. The parameters and the associated level for the optimal model formulation are molecular weight of PVA = 89,00Da (high), concentration of PLGA = 20 w/v % (medium), organic/aqueous phase ratio = 1:1 (medium), vortexing speed = 9 (high), vortexing duration = 60 seconds (low), stirring speed for hardening process = 1200 rpm (high) and solvent evaporation duration = 24 h (medium).

## Conclusion

In this study, Taguchi OA design proved to be a valuable tool in the optimization of several processing parameters in solvent evaporation technique with complex interrelationship with few experiments. The design was efficient for identifying the parameters which had significant effect on microparticles size More precisely, vortexing speed, concentration of PLGA, organic/aqueous phase ratio, stirring speed, concentration of PVA in primary emulsion, vortexing duration were significant whereas the molecular weight of PVA and concentration of PVA in hardening bath were proven to be not important parameters with regard to PLGA microparticle size. The optimal model formulation was established as molecular weight of PVA = 89,00 Da, concentration of PLGA = 20 w/v %, organic/aqueous phase ratio = 1:1, vortexing speed = 9, vortexing duration = 60 s, stirring speed for hardening process = 1200 rpm and solvent evaporation duration = 24 h. These optimum levels of the parameters were useful in the fabrication of PLGA microparticles with the minimum particle size of 23.51 μm. A model drug (BSA) was successfully incorporated into the optimised microparticles, which had no statistically significant impact on size.
